# Prediction of recurrent stroke with ABCD2 and ABCD3 scores in patients with symptomatic 50-99% carotid stenosis

**DOI:** 10.1186/s12883-014-0223-y

**Published:** 2014-11-30

**Authors:** Elias Johansson, Jakob Bjellerup, Per Wester

**Affiliations:** Pharmacology and Clinical Neuroscience, Umeå University, Umeå, Sweden; Public Health and Clinical Medicine, Umeå University, Umeå, Sweden

**Keywords:** Stroke, Carotid stenosis, Risk, ABCD2, ABCD3

## Abstract

**Background:**

Although it is preferable that all patients with a recent Transient Ischemic Attack (TIA) undergo acute carotid imaging, there are centers with limited access to such acute examinations. It is controversial whether ABCD2 or ABCD3 scores can be used to triage patients to acute or delayed carotid imaging. It would be acceptable that some patients with a symptomatic carotid stenosis are detected with a slight delay as long as those who will suffer an early recurrent stroke are detected within 24 hours. The aim of this study is to analyze the ability of ABCD2 and ABCD3 scores to predict ipsilateral ischemic stroke among patients with symptomatic 50-99% carotid stenosis.

**Methods:**

In this secondary analysis of the ANSYSCAP-study, we included 230 consecutive patients with symptomatic 50-99% carotid stenosis. We analyzed the risk of recurrent ipsilateral ischemic stroke before carotid endarterectomy based on each parameter of the ABCD2 and ABCD3 scores separately, and for total ABCD2 and ABCD3 scores. We used Kaplan-Meier analysis.

**Results:**

None of the parameters in the ABCD2 or ABCD3 scores could alone predict all 12 of the ipsilateral ischemic strokes that occurred within 2 days of the presenting event, but clinical presentation tended to be a statistically significant risk factor for recurrent ipsilateral ischemic stroke (p = 0.06, log rank test). An ABCD2 score ≥2 and an ABCD3 score ≥4 could predict all 12 of these strokes as well as all 25 ipsilateral ischemic strokes that occurred within 14 days. To use ABCD3 score seems preferable over the ABCD2 score because a higher proportion of low risk patients were identified (17% of the patients had an ABCD3 score <4 while only 6% had an ABCD2 < 2).

**Conclusions:**

Although it is preferable that carotid imaging be performed within 24 hours, our data support that an ABCD3 score ≥4 might be used for triaging patients to acute carotid imaging in clinical settings with limited access to carotid imaging. However, our findings should be validated in a larger cohort study.

## Background

Since its introduction in 2005 [1], the ABCD-score has been thoroughly validated [2]. In large cohorts (n > 1 000), the score has been amended and validated with parameters readily available in the emergency department, namely diabetes [3,4] and dual events [4,5], and with those requiring investigations, namely cerebral infarction on Computed Tomography or Magnetic Resonance Imaging [4-6] and the presence of an ipsilateral ≥50% carotid stenosis [4,5]. It has also been proposed to amend the score based on large-artery atherosclerosis instead of an ipsilateral ≥50% carotid stenosis [4]. In smaller cohorts (n < 300), the addition of C-reactive protein [7], D-dimer [8], hypertension [9], and hyperglycemia [9] have been proposed but not validated. The validated amendments have increased the ability of the scores to discriminate risk: The ABCD2 score with diabetes is superior to the ABCD score [3], the ABCD3 score with dual events is superior to the ABCD2 score [5], and the ABCD3I score with infarction on Computed Tomography and Magnetic Resonance Imaging and the presence of an ipsilateral ≥50% carotid stenosis is superior to the ABCD3 score [5].

The exact role of the ABCD-type score in the management of patients is unclear. One controversial issue is whether ABCD-type scores can be used to triage patients to acute or delayed carotid imaging [5,8,10-15]. In clinical settings where carotid imaging is available (directly or by transfer), it has been proposed that all patients with Transient Ischemic Attack (TIA) should undergo acute carotid imaging within 24 hours [5]. However, at least in our experience, many clinical settings have limited access to immediate carotid imaging and acute transfer to centers with acute carotid imaging is not feasible because of long distances and high costs. In such clinical settings, there is a need for triaging patients to acute or delayed carotid imaging.

Several studies have shown that 31-48% of patients with TIA or minor stroke with carotid stenosis have an ABCD2-score <4 points, questioning the usefulness of the ABCD2-score for triaging carotid imaging [14-17]. However, ABCD-type scores do not have to detect all patients with symptomatic carotid stenosis as long as they identify the patients with symptomatic carotid stenosis who will suffer a subsequent ipsilateral ischemic stroke. Only one study has analyzed the ability of ABCD2 to predict recurrent strokes (unspecified if ischemic or ipsilateral) among patients with symptomatic carotid stenosis, but this study was limited by a small sample size (n = 29 and 2 strokes) [18].

The aim of this study is to analyze the ability of the ABCD2 and ABCD3 scores to predict recurrent ipsilateral ischemic stroke among patients with symptomatic 50-99% carotid stenosis.

## Methods

### Patients

This is a secondary analysis of the Additional Neurological SYmptoms before Surgery of the Carotid Arteries – a Prospective study (ANSYSCAP) [19]. To date, the ANSYSCAP study was the largest single-center study of patients with 50-99% carotid stenosis with up-to-date medical prevention [19]. In short, the ANSYSCAP study prospectively included 230 consecutive patients with symptomatic 50-99% carotid stenosis. The patients were included at the Umeå Stroke Center, Sweden, between 2007-08-01 and 2009-12-31. The sample size was derived from setting study start- and stop dates. Most patients (81%) were sent from 11 referring hospitals. We only included patients that were preliminarily eligible for carotid endarterectomy (CEA). This was defined as a patient who underwent a pre-operative evaluation aimed at CEA. Since this was a selected population, the CEA rate was high (80%). Reasons for not performing CEA were low predicted benefit (7%), too high perioperative risk due to co-morbidities or technical reasons (5%), CEA not meaningful (4%) or patient refusal (4%). Over the course of the study, CEA went from being most often scheduled to be performed earlier. Whenever possible, the delay to CEA was kept as short as possible. The observation period for recurrent stroke was the first 90 days after the presenting event in the primary analysis. For the majority of patients that underwent CEA within 90 days, the observation time was limited to up to the CEA. In order to reduce a possible bias caused by non-random censoring at CEA, the current analysis was limited to the first 14 days after the presenting event. The baseline characteristics of patients are shown in Table 1. In the current analysis, we included both patients with a presenting event lasting <24 hours (TIA and amaurosis fugax) and ≥24 hours (stroke and retinal artery occlusion) because our sample size was small.

**Table 1:** **Baseline patient data**

	**All (n = 230)**
Men n (%)	147 (64%)
Age, years mean (SD)	71 (7.7)
50-69% symptomatic carotid stenosis n (%)	54 (23%)
70-99% symptomatic carotid stenosis n (%)	176 (77%)
First recorded systolic blood pressure mean (SD)	160 (29)
First recorded diastolic blood pressure mean (SD)	82 (16)
Presenting event: Stroke n (%)	96 (42%)
Presenting event: Retinal artery occlusion n (%)	12 (5%)
Presenting event: TIA n (%)	70 (30%)
Presenting event: Amaurosis fugax n (%)	52 (23%)
Anti-platelet or anti-coagulant at first health care contact n (%)	130 (57%)
Anti-platelet or anti-coagulant at 2 days after first health care contact n (%)	195 (85%)
Anti-platelet or anti-coagulant at 7 days after first health care contact n (%)	214 (93%)
Blood-pressure lowering medication at first health care contact n (%)	189 (82%)
Blood-pressure lowering medication at 2 days after first health care contact n (%)	198 (86%)
Blood-pressure lowering medication at 7 days after first health care contact n (%)	205 (89%)
Lipid lowering medication at first health care contact n (%)	99 (43%)
Lipid lowering medication at 2 days after first health care contact n (%)	142 (62%)
Lipid lowering medication at 7 days after first health care contact n (%)	176 (77%)
Underwent CEA or carotid angioplasty n (%)	183 (80%)
Delay between presenting event and first health care contact, days median (IQR)	0 (0-2)
Delay between presenting event and CEA or carotid angioplasty, weeks median (IQR)	4.1 (2.4-7.1)
CEA or carotid angioplasty within 2 days of the presenting event	1 (0.4%)
CEA or carotid angioplasty within 7 days of the presenting event	10 (4%)
CEA or carotid angioplasty within 14 days of the presenting event	36 (16%)
Recurrent ipsilateral ischemic stroke n (%)	25 (11%)

### Data acquisition

In the original study, blood pressure was recorded at the time of the pre-operative evaluation. The first blood pressure should be used for the ABCD2 and ABCD3 scores. Therefore, we retrospectively amended our data with the first recorded blood pressure. This was done by a review of medical records. We recorded the patient’s first documented blood pressure – most often at the first health care contact. The remaining variables of the ABCD2 and ABCD3 scores were collected in the original study.

### Calculation of ABCD2 and ABCD3 scores

We calculated all variables for the ABCD2 and ABCD3 scores as defined elsewhere: [5] (A) Age <60 years 0 points and ≥60 years 1 point; (B) first recorded blood pressure <140/90 0 points and either ≥140 systolic or ≥90 diastolic 1 point; (C) clinical features of the presenting event with focal weakness 2 points, speech impairment without weakness 1 point, and other 0 points; (D1) duration of presenting event <10 minutes 0 points, 10-59 minutes 1 point, and ≥60 minutes 2 points; (D2) diabetes absent 0 points and present 1 point; and (D3) for ABCD3-scores only, dual events with ≥1 additional TIA or amaurosis fugax within 7 days of the presenting event 2 points and no such event 0 points. All patients with stroke or retinal artery occlusion as the presenting event were assigned 2 points for duration.

### Endpoint & analyses

We used the same endpoint as in the original ANSYSCAP study: Recurrent ipsilateral ischemic stroke. Ipsilateral retinal artery occlusion was also included in the endpoint (and is henceforth included in “ipsilateral ischemic stroke”). We analyzed recurrent ipsilateral stroke within 14 days after the presenting event. If the patient underwent CEA within 14 days, we only analyzed events that occurred before the CEA (excluding all perioperative events). The presenting event was defined as the last ischemic cerebrovascular event (stroke, retinal artery occlusion, TIA, or amaurosis fugax) before the patient sought health care.

We analyzed the risk of the endpoint at 2 days, 7 days, and 14 days. We analyzed the risk of the endpoint for each parameter in the ABCD2 and ABCD3 scores separately and for different total ABCD2 and ABCD3 scores. We analyzed an ABCD2 score of 2, 3, and 4 points as cut-offs for high and low risk. We used the same cut-offs for the ABCD3 score as in a previous study [8]: low (0-3 points), moderate (4-5 points), and high (6-9 points) risk.

### Ethics

The local Ethical Review Board found the study to be completely observational and therefore did not incur any ethical problems and did not require ethics approval.

### Statistics

We calculated the 14-day risk of recurrent ipsilateral ischemic stroke with Kaplan-Meier curves. We used CEA as a censor and, therefore, patients that underwent CEA only contributed risk-time before their CEA and all perioperative events were excluded. The risks at 2, 7, and 14 days were acquired from this survival analysis. We used the log rank test for differences between groups. A p-value <0.05 was defined as statistically significant. We used IBM SPSS 20.0 Statistical software for all calculations.

## Results

Data for age, first recorded blood pressure, clinical presentation, diabetes, and dual events were available for all 230 cases. Five cases were excluded from analyses of the event duration and total ABCD2 and ABCD3 scores because the duration of the presenting event could only be determined as less than 24 hours. The mean observation time was 12.3 (SD 3.6) days, 179 (78%) were observed for 14 days.

When analyzing the parameters in the ABCD2 and ABCD3 scores separately, only clinical presentation (focal weakness, speech disturbance, or other focal neurological deficit) tended to be a statistically significant risk factor for recurrent ipsilateral ischemic stroke (p = 0.06, log rank test), see Table 2. Not one of the six parameters was present in all 12 patients that suffered a recurrent ischemic stroke within 2 days. A ≤3% risk of recurrent ipsilateral ischemic stroke at 2 days was observed among the patients without clinical presentation of focal weakness or speech disturbance and the patients with a presenting event lasting <10 minutes.

**Table 2:** **Risk of recurrent ipsilateral ischemic stroke at 2, 7, and 14 days after the presenting event for each of the parameters in the ABCD3 score**

	**Pat**	**2 days**		**7 days**		**14 days**		**P-value**
		**n**	**Risk**	**n**	**Risk**	**n**	**Risk**	
Age <60 years	19	1	5% (0-15%)	1	5% (0-15%)	2	11% (0-25%)	p = 0.95
Age ≥60 years	211	11	5% (2-8%)	17	8% (4-12%)	23	11% (7-16%)	
Blood Pressure <140/90	46	5	11% (2-20%)	7	15% (5-26%)	8	17% (6-28%)	p = 0.10
Blood Pressure ≥140/90	184	7	4% (1-7%)	11	6% (2-10%)	17	10% (5-14%)	
Clinical presentation: Other	64	2	3% (0-7%)	2	3% (0-7%)	2	3% (0-7%)	p = 0.06
Clinical presentation: Speech disturbance	17	1	6% (0-17%)	3	18% (0-37%)	3	18% (0-37%)	
Clinical presentation: Focal weakness	149	9	6% (2-10%)	13	9% (4-13%)	20	14% (8-20%)	
Duration <10 min*	59	2	3% (0-8%)	2	3% (0-8%)	4	7% (0-14%)	p = 0.45
Duration 10-59 min*	36	2	6% (0-13%)	5	14% (3-25%)	5	14% (3-25%)	
Duration ≥60 min*	130	8	6% (2-10%)	11	9% (4-13%)	16	13% (7-18%)	
Diabetes: Absent	168	9	5% (2-9%)	13	8% (4-12%)	18	11% (6-16%)	p = 0.91
Diabetes: Present	62	3	5% (0-10%)	5	8% (1-15%)	7	12% (3-20%)	
Dual events: Absent	183	10	5% (2-9%)	14	8% (4-12%)	19	11% (6-15%)	p = 0.61
Dual events: Present	47	2	4% (0-10%)	4	9% (0-17%)	6	14% (3-25%)	

*5 cases excluded because of unknown duration of the presenting event.

Risk data derived from Kaplan-Meier analyses using CEA as a censor.

**Figure 1:**
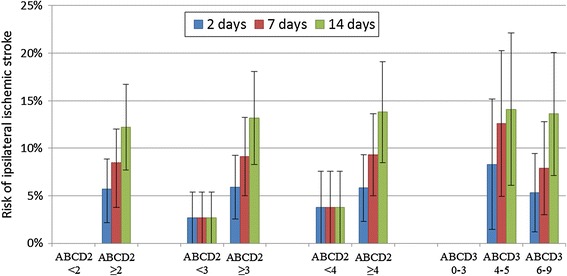
**Risks of recurrent ipsilateral ischemic stroke for different cut-off ABCD2 and ABCD3 scores.** Risk data derived from Kaplan-Meier analyses using CEA as a censor. Error bars denotes 95% CI.

The risks of recurrent ipsilateral ischemic stroke for different ABCD2 and ABCD3 scores are presented in Table 3. Ninety-four percent of the patients had an ABCD2 score ≥2 points, 84% had ≥3 points, and 76% had ≥4 points. Seventeen percent of the patients had an ABCD3 score of 0-3 points, 64% had 4-6 points, and 19% had 7-9 points. Of the 25 patients that suffered a recurrent ipsilateral ischemic stroke within 14 days, 100% had an ABCD2 score ≥2 points, 96% had ≥3 points, and 92% had ≥4 points; while for ABCD3 scores, 0% had 0-3 points, 84% had 4-6 points, and 16% had 7-9 points. The risks of recurrent ipsilateral ischemic stroke for the cut-off ABCD2 and ABCD3 scores are depicted in Figure 1. We analyzed if the risk of ipsilateral ischemic stroke differed between various cut-offs for ABCD2 and ABCD3 scores. We found that the risk of recurrent ipsilateral ischemic stroke tended to be statistically significant for the ABCD2 cut-offs of ≥3 points (p = 0.08) and ≥4 points (p = 0.06) and the ABCD3 categories 0-3, 4-5, and 6-9 points (p = 0.052), but not for the ABCD2 cut-off of ≥2 points (p = 0.19, log rank test).

**Table 3:** **Risk of recurrent ipsilateral ischemic stroke at 2, 7, and 14 days after the presenting event based on ABCD2 and ABCD3 scores**

**ABCD2**	Pat (%)*	**2 days**		**7 days**		**14 days**	
		**n**	**Risk (95% CI)**	**n**	**Risk (95% CI)**	**n**	**Risk (95% CI)**
0-1p	13 (6%)	0	-	0	-	0	-
2p	24 (11%)	1	4% (0-12%)	1	4% (0-12%)	1	4% (0-12%)
3p	16 (7%)	1	6% (0-18%)	1	6% (0-18%)	1	6% (0-18%)
4p	38 (17%)	4	11% (1-20%)	5	13% (2-24%)	7	19% (6-32%)
5p	36 (16%)	0	-	3	9% (0-18%)	4	11% (1-22%)
6p	71 (32%)	5	7% (1-13%)	7	10% (3-17%)	10	14% (6-22%)
7p	27 (12%)	1	4% (0-11%)	1	4% (0-11%)	2	8% (0-19%)
**ABCD3**	Pat (%)*	**2 days**		**7 days**		**14 days**	
		**n**	**Risk (95% CI)**	**n**	**Risk (95% CI)**	**n**	**Risk (95% CI)**
0-3p	39 (17%)	0	-	0	-	0	-
4p	35 (16%)	5	14% (3-26%)	6	17% (5-30%)	6	17% (5-30%)
5p	37 (16%)	1	3% (0-8%)	3	8% (0-17%)	4	11% (1-21%)
6p	73 (32%)	5	7% (1-13%)	6	8% (2-15%)	11	16% (7-24%)
7p	30 (13%)	1	3% (0-10%)	2	7% (0-16%)	3	11% (0-22%)
8p	8 (4%)	0	-	1	13% (0-35%)	1	13% (0-35%)
9p	3 (1%)	0	-	0	-	0	-

*5 cases excluded because of unknown duration of the presenting event.

Risk data derived from Kaplan-Meier analyses using CEA as a censor.

## Discussion

Among the patients with symptomatic 50-99% carotid stenosis included in this study, 25 suffered a recurrent ipsilateral ischemic stroke within 14 days and all of these patients had an ABCD3 score ≥4 points. The overall approach is the main novelty of this article – although ABCD-type score cannot detect a sufficient share of the patients with carotid stenosis, they seem to be able to detect a sufficient share of the patients with carotid stenosis that suffer an early recurrent ipsilateral ischemic stroke. However, due a small sample size, no firm conclusions can be drawn.

None of the other parameters could predict all the recurrent ipsilateral strokes that occurred within 2 days. However, both an ABCD2 score ≥2 points and an ABCD3 score ≥4 points were able to predict all events at 2 days as well as at 14 days. There were more patients without recurrent strokes with ABCD3 scores <4 points (17%) than ABCD2 scores <2 points (6%); therefore, it is reasonable to regard the ABCD3 score as clinically more useful for triaging to acute or delayed carotid imaging. Similarly, in TIA patients with mixed etiologies, 8.0% (208/2611) have an ABCD2 score <2 points and 33% (802/2445) have an ABCD3 score <4 points (Personal communication with PJ Kelly and Á Merwick in 2013 regarding [5]). In addition, the ABCD2 cut-off of <2 points was non-significant, but this is likely a false negative finding caused by a low number (n = 13) of patients with <2 points.

Our data confirm the findings of previous studies that an ABCD2 score <4 points does not detect all patients with carotid stenosis [8,14-17]. Since a symptomatic carotid stenosis incurs a high risk of stroke recurrence, we concur with Merwick and colleagues [5] that carotid imaging should generally be performed within 24 hours in all TIA/minor ischemic stroke patients eligible for CEA. It may not be meaningful to triage patients to delayed carotid imaging in clinical settings with general 24/7 access to acute carotid imaging; although, a cost-benefit analysis is warranted. However, it is our experience that there are clinical settings (small rural hospitals) with limited access to carotid imaging (whether the patient is admitted or not) and too long distances for regular transfers. In such settings there is a need for an evidence based way to triage patients to carotid imaging. In such settings, it is likely that this will incur benefit rather than harm because the patient with the highest risk is examined first. Our results points to that an ABCD3 score of <4 can be used for this triage. Since an ABCD3 score <4 is quite rare (17%) among patients with carotid stenosis, one could question if such a triage system would be meaningful. However, such a triage system would be meaningful because in the intended population (patients with TIA of mixed etiologies), an ABCD3 score of <4 is more common – 33% (Personal communication with PJ Kelly and Á Merwick in 2013 regarding [5]).

The risk reducing effect of CEA seems to occur within the first 3 years [20]. Therefore, although our 2, 7, and 14 day risks might be used for triage before CEA, it would be inappropriate to base the decision to perform CEA on our results.

Several aspects of the current study design should be considered. The issue of timing in carotid imaging is mainly of interest for patients that might be eligible for CEA and we only included patients who were preliminarily eligible for CEA. In addition, our outcome was ipsilateral ischemic stroke, which is the serious outcome most likely to be preventable by early CEA. Since the issue of the timing of carotid imaging is focused on the risk before CEA, we censored patients after they underwent CEA. There are also weaknesses in this study. A few patients with a major recurrent stroke that occurred before they were referred to the Umeå Stroke Center were likely missed. The results are not valid for all patients with symptomatic carotid stenosis because we excluded those not preliminarily eligible for CEA because of major stroke as the presenting event, advanced age, or serious co-morbidities. Although much larger than the previous similar study [18], the sample size (n = 230 with 25 strokes) was small and, therefore, we were not able to analyze patients with presenting events lasting <24 hours alone. This is a small hypothesis generating study and the findings in this study need to be validated. This is likely achievable by re-analyzing the cohorts with carotid stenosis as a risk factor rather than analyzing patients with carotid stenosis separately [4,5,21,22].

## Conclusion

Although some patients with symptomatic 50-99% carotid stenosis had low ABCD2 and ABCD3 scores, none of these patients suffered a recurrent ipsilateral ischemic stroke before CEA. Although our findings need to be validated and confirmed in further studies, our results suggest an ABCD3 score of 0-3 or ≥4 might be used for triaging patients to delayed or acute carotid imaging in clinical settings with limited access to carotid imaging.

## Abbreviations

ANSYSCAP: Additional Neurological SYmptoms before Surgery of the Carotid Arteries – a Prospective study
CEA: Carotid end arterectomy
TIA: Transient ischemic attack
